# Survey of factors influencing women’s selection of the delivery method of their second child in Shanxi Province, China

**DOI:** 10.12669/pjms.37.3.2634

**Published:** 2021

**Authors:** Jin-qiong Li

**Affiliations:** 1 Jin-qiong Li Department of Obstetrics and Gynecology, Heji Hospital Affiliated to Changzhi Medical College, Changzhi, Shanxi, 046000, P. R. China

**Keywords:** Second child, Delivery method, Influencing factor

## Abstract

**Objective:**

To explore the factors influencing women’s selection of the delivery method of their second child.

**Methods:**

A questionnaire survey was administered among 431 women in the age range of interest from January 2015 to January 2017, and the survey results were evaluated and analyzed statistically. The experts evaluating the questionnaire are professionals in the Department of Obstetrics and Gynecology, Heji Hospital Affiliated to Changzhi Medical College.

**Results:**

A total of 70.99% of subjects were 28-35 years old. Approximately 82.35% wished to undergo vaginal delivery, and the remaining 17.65% expressed to undergo cesarean delivery. The reasons for cesarean delivery included the following: fetal factors: worry about fetal health (33.33%), birth trauma (12.90%), and fetal macrosomia (38.17%); maternal factors: advanced age (36.56%), inability to bear uterine contraction pains (21.51%), worry about uracratia after vaginal delivery (10.75%), worry about perineum laceration (8.60%) and the impacts on sexual gratification after delivery (5.38%); social factors: faster delivery mode (54.84%), selection of birth time (27.96%), and better planning of maternity leave (17.20%).

**Conclusion:**

Most women tend to undergo vaginal delivery. However, due to the influence of age, educational level and other factors, an increasing number of women prefer cesarean delivery. Medical institutions have the responsibility for providing overall and fair medical information to women of childbearing age to help them make informed choices regarding mode of delivery.

## INTRODUCTION

Delivery is an important event in women’s lives. As the two-child policy is implemented in China, an increasing number of women of reproductive age, including older women, choose to have a second child. Many elect to have cesarean delivery. The factors influencing women’s second-child delivery method are explored in this study. We wished to provide the best delivery plan based on the actual situation of the mother in the future.

In a traditional sense, it is believed that vaginal delivery is the most natural delivery method. Since cesarean delivery involves operation and anesthesia risks as well as many intraoperative and postoperative complications, it is considered a risky delivery method. In recent years, with the development of medical reproductive technology, many patients who were originally infertile can become pregnant. The probability of fertilization of a “precious child” increases, and such women pursue a safe delivery mode with a short delivery process. Thus, the proportion of cesarean delivery increases greatly.^1^ In addition, people’s attitudes toward cesarean delivery has also changed. For example, the desire for a lucky birth time and more effective planning for maternity leave have increased the trend of cesarean section before regular uterine contractions without medical indications (i.e., cesarean section resulting from social factors).^2^

The decision to have cesarean delivery or vaginal delivery is closely related to the opinions of the women. There are many factors influencing women’s selection of delivery method, such as the bad childbirth experience, concerns about the health of the mother and baby, the pain of childbirth during uterine contractions, and the description of the pain about childbirth by relatives and friends. In China, after the introduction of the second-child policy, the main reasons why women choose cesarean section include the unforgettable painful experience of the first child, the advanced age that is not suitable for vaginal delivery, etc.^3^ Thus, cesarean delivery has become the preferred delivery method. Current data show that the cesarean delivery rate presents a rising trend. Martin JA et al. reported that the cesarean section rate had risen to 29.1% in 2004, and to 32% in 2017.^4^,^5^ In 1998, the cesarean section rates in urban and rural areas of China were 19.9% and 3.6%, respectively, and in 2008, they had risen to 54.1% and 23.6%, respectively.^6^ This study aims to analyze the factors influencing women’s selection of mode of delivery,so as to provide data support for suggesting the best delivery plan for pregnant women.

## METHODS

This study is a retrospective descriptive study based on survey method. The study was approved by the Institutional Ethics Committee of Heji Hospital Affiliated to Changzhi Medical College, and written informed consent was obtained from all participants.

### Inclusion criteria:

1. Women during pregnancy; 2. Those between 18-45 years old; 3. Those who have conceived one or more children.

### Exclusion criteria:

1. Pregnant women with chronic diseases such as hypertension or diabetes; 2. Those younger than 18 years old or older than 45 years old; 3. Those with mental illness who cannot decide on their own. Four hundred forty-two women of childbearing age who were treated in the Department of Obstetrics and Gynecology, Heji Hospital Affiliated to Changzhi Medical College were included in the study. Those who received prenatal check-ups from January 2015 to January 2017 were chosen, and 436 women agreed to participate in this study. A total of 436 women were asked to answer the questions on the questionnaire. Informed consent was obtained from the 436 women, and they signed the informed consent form. A total of 431 questionnaires were received, with a recovery rate of 98.85%. All participants completed the questionnaire anonymously. Five questionnaires were not completed in full, so they were excluded. Therefore, 431 completed questionnaires were analyzed (as shown in Table-I). All data were kept confidential, and only the researchers investigated the data. It only discusses the opinions of subjects on the delivery mode and fails to conduct research tracking their final delivery method.

**Table-I:** Demographic characteristics of women in the childbearing period (n=431).

	No. (n=431)	Percentage 100(%)	Selection of vaginal delivery (n=338)	Percentage 78.42(%)	Selection of cesarean delivery (n=93)	Percentage 21.58(%)
***Age (years)***						
18-27	48	11.14	34	70.83	14	29.17
28-35	306	70.99	252	82.35	54	17.65
35-45	77	17.87	52	67.53	25	32.47
mean age	33.29±3.08		33.83±2.86		33.11±2.17	
***Birthplace***						
Urban area	187	43.39	156	83.42	31	16.58
Rural area	244	56.61	182	74.59	62	25.41
***Place of residence***						
Urban area	202	46.87	140	69.31	62	30.69
Rural area	229	53.13	198	86.46	31	13.54
***Educational level***						
Higher education	102	23.67	73	71.57	29	28.43
Secondary education	134	31.09	108	80.60	26	19.40
Primary school and below	195	45.24	157	80.51	38	19.49
***Employment status***						
Housewife	242	56.15	186	76.86	56	23.14
Part-time job	64	14.85	50	78.13	14	21.87
Full-time job	25	29.00	102	81.60	23	18.40
***Family income***						
Above 8000 yuan	98	22.74	73	74.49	25	25.51
3000-8000 yuan	112	25.99	93	83.04	19	16.96
Below 3000 yuan	221	51.28	172	77.83	49	22.17
***Delivery information source***						
Relatives and friends	192	44.55	138	71.88	54	28.12
Personal experience	162	37.59	128	79.01	34	20.99
Books and network	77	17.86	72	93.51	5	6.49
Total	431		338		93	

**Table-II:** Factors influencing delivery mode.

	Selection of vaginal delivery (n=338)	Percentage 78.42(%)	Selection of cesarean delivery (n=93)	Percentage 21.58(%)
***Fetal factors***		
Newborn health	192	56.80	31	33.33
Newborn birth trauma	116	34.32	12	12.90
Fetal macrosomia	18	5.33	36	38.71
Twins/triplets	12	3.55	14	15.05
***Maternal factors***		
Maternal health	128	37.87	16	17.20
Elderly parturient woman	68	20.12	34	36.56
Fear of uterine contraction pain	47	13.90	20	21.51
Abdominal wall scarring	26	7.69	0	0
Worry about perineum laceration	35	10.36	8	8.60
Worry about uracratia after vaginal delivery	26	7.69	10	10.75
Postpartum sexual satisfaction	8	2.37	5	5.38
***Social factors***		
Natural delivery	320	94.67	0	0
Faster delivery mode	12	3.55	51	54.84
Better planning for annual leave	6	1.78	16	17.20
Selecting birth time	0	0	26	27.96

### Investigation Methods:

Four hundred thirty-six subjects in the study were investigated and were required to answer the questions on the questionnaire. Five questionnaires were not fully completed, so they were rejected. After the remaining 431 questionnaires were received, professional researchers evaluated them, and they all met the requirements. A total of 436 questionnaires were received anonymously. The researchers evaluating the questionnaires included one obstetrician, one nurse from the obstetrics department and one midwife. The three experts were responsible for assessing the quality of all questionnaires. The content of the questionnaire mainly consisted of two parts: participants’ social demographic data survey and a survey on the information source of women’s preferred delivery method and influencing factors.

### Statistical Analysis Method:

Excel 2007 was used to establish a database, and SPSS 13.0 was applied to analyze the database.

## RESULTS

Among the 431 research subjects, the number of people between 28-35 years old was the largest, 306 cases (70.99%), with an average age of 31.17±2.66 years. Among them, 252 women (82.35%) tend to give birth naturally, and 54 women (17.65%) were willing to have a cesarean section. In the other two age groups, the percentage of those who chose vaginal delivery was also higher than that of those who chose cesarean section. Among the subjects, 202 women (46.87%) lived in urban areas, and 140 of them (69.31%) choose vaginal delivery. There were 229 women (53.13%) living in rural areas, of which 198 people (86.46%) choose vaginal delivery. There were 102 women (23.67%) who have received higher education, of which 73 women (71.57%) were willing to give birth vaginally. The percentage of those who received secondary education or elementary school education who chose vaginal delivery were also higher than those who chose cesarean section (Table-I).

Among 93 women who wanted to undergo cesarean delivery, 31 worried about fetal health (33.33%); 12 worried about newborn birth trauma (12.90%); 36 preferred cesarean delivery due to fetal macrosomia (38.17%); and 12 chose cesarean delivery due to multiple pregnancy (15.05%). A total of 36.56% wished to undergo cesarean delivery because of maternal elements. The other reasons included the inability to bear uterine contraction pain (21.51%), maternal self-care (17.20%), worry about perineum laceration (8.60%) and the impacts on sexual gratification after delivery (5.38%). Social factors are also a common cause of cesarean delivery, such as selecting a faster delivery method (54.84%), choosing the time of birth (27.96%) and planning more effectively for maternity leave (17.20%).

## DISCUSSION

In this study, most women of childbearing age were between 28 and 35 years, and the distribution of these subjects in urban areas and rural areas was almost similar, i.e., 46.87% and 53.13%, respectively. Thus, the sample in this study is representative of women of childbearing age in the area where the study was performed. In this study, the proportion of women preferring vaginal delivery (338, 78.42%) exceeded the proportion of women preferring cesarean delivery (93, 21.57%). The proportion of urban women who preferred vaginal delivery was 68.98% (140 cases), which was slightly lower than that of rural women who preferred vaginal delivery (198, 74.59%).

The results of this study show that age, educational level and occupation were significantly correlated with women’s selection of different delivery methods. Elderly parturient women were more likely to choose cesarean delivery because they were concerned with the risks to the fetuses and themselves due to frequent uterine contractions in the process of vaginal delivery. Moreover, elderly parturient women may have a (higher) chance of suffering complications. Hence, elderly parturient women consider cesarean delivery to be a safer delivery method. Such delivery modes can effectively lower the incidence of adverse outcomes. Data shows that the fertility rate of Chinese pregnant and parturient women aged between 25-29 year old has reduced to about 96%, while the fertility rate has increased to about 17% in women at the age of 35-39 and increased to about 5.74% in women at the age of 40-44.^7^,^8^

In terms of educational level, the proportion of women with a high educational level selecting cesarean delivery is greater than that of women with a low educational level selecting cesarean delivery. This may be related to the following factors: the women with a high level of educational have many channels of receiving knowledge on vaginal delivery and worry about the safety of the fetuses and themselves in the long-term vaginal delivery process. In addition, such women are concerned with the side effects of vaginal delivery, such as perineal lacerations, the decline in sexual gratification caused by vaginal relaxation, and urethral leakage after delivery. Mumtaz S et al. have also reported that women with higher education and urban life are more likely to have a caesarean section.^9^ Furthermore, some women require propitious birth time and better planning of annual leave so that the probability of selecting cesarean delivery increases. These reasons are more common among contemporary women who have their own professions. The research of He Yiwen et al. also indicates that the decline in the vaginal delivery rate of elderly parturient women and the rise in the cesarean section rate are based on specific medical reasons.^10^

The women preferring vaginal delivery possibly consider this to be “the most natural delivery process”. Among this group of women, most consider vaginal delivery to allow them to leave the hospital earlier and shorten the length of hospital stay. Moreover, they can recover faster and engage in breastfeeding earlier without undergoing unnecessary operation and anesthesia risks or abdominal wall scarring after the operation. For women preferring cesarean delivery, the most common reason for choosing such a delivery mode is to avoid labour pains. Meanwhile, they are concerned that the fetus suffers long-term damage from the delivery process. Labour pains are considered to be one of the most painful events in the human experience, and women fear such pain in the delivery process.^11^ The results show that the women selecting cesarean delivery are greatly influenced by the suggestions of medical staff. It has been reported that some medical workers hold a negative attitude toward vaginal delivery,^12^ therefore, they encourage women to select cesarean delivery to protect their pelvic floor tissue function and reduce the risk of uracratia or fecal incontinence.^13^

### Limitations of the study.

Future studies are required to further follow up and determine the final delivery method selected by pregnant women after investigating their willingness to give birth. In this study, the rate of vaginal delivery tended to increase, but there is evidence showing that an increasing number of women express their preference for cesarean delivery.^14^ Although the age and educational level of women largely influence their selection of delivery mode, the view of professional medical workers also plays an important role in the selection of the delivery mode, indicating that medical institutions have the responsibility of offering overall and fair medical information to women of childbearing age to help them make informed choices regarding delivery mode.

### Authors’ Contributions:

**JQL:** Designed this study, prepared this manuscript, and is responsible and accountable for the accuracy of the work.
